# Identification and validation of ferroptosis key genes in bone mesenchymal stromal cells of primary osteoporosis based on bioinformatics analysis

**DOI:** 10.3389/fendo.2022.980867

**Published:** 2022-08-25

**Authors:** Yu Xia, Haifeng Zhang, Heng Wang, Qiufei Wang, Pengfei Zhu, Ye Gu, Huilin Yang, Dechun Geng

**Affiliations:** ^1^ Department of Orthopedics, The First Affiliated Hospital of Soochow University, Suzhou, China; ^2^ Department of Orthopedics, Changshu Hospital Affiliated to Soochow University, First People’s Hospital of Changshu City, Changshu, China

**Keywords:** primary osteoporosis, ferroptosis, bioinformatics, bone mesenchymal stromal cells (BMSCs), autophagy

## Abstract

Primary osteoporosis has long been underdiagnosed and undertreated. Currently, ferroptosis may be a promising research direction in the prevention and treatment of primary osteoporosis. However, the specific mechanism of ferroptosis in primary osteoporosis remains a mystery. Differentially expressed genes (DEGs) were identified in bone mesenchymal stromal cells (BMSCs) of primary osteoporosis and heathy patients from the GEO databases with the help of bioinformatics analysis. Then, we intersected these DEGs with the ferroptosis dataset and obtained 80 Ferr-DEGs. Several bioinformatics algorithms (PCA, RLE, Limma, BC, MCC, etc.) were adopted to integrate the results. Additionally, we explored the potential functional roles of the Ferr-DEGs *via* GO and KEGG. Protein–protein interactions (PPI) were used to predict potential interactive networks. Finally, 80 Ferr-DEGs and 5 key Ferr-DEGs were calculated. The 5 key Ferr-DEGs were further verified in the OVX mouse model. In conclusion, through a variety of bioinformatics methods, our research successfully identified 5 key Ferr-DEGs associated with primary osteoporosis and ferroptosis, namely, sirtuin 1(*SIRT1*), heat shock protein family A (*Hsp70*) member 5 (*HSPA5*), mechanistic target of rapamycin kinase (*MTOR*), hypoxia inducible factor 1 subunit alpha (*HIF1A*) and beclin 1 (*BECN1*), which were verified in an animal model.

## Introduction

As a complex bone metabolism disorder mainly characterized by bone loss and bone microstructure changes, primary osteoporosis has long been underdiagnosed and undertreated (1–4). Approximately 40% of postmenopausal white women are affected by osteoporosis. The aging population will exacerbate the medical and socioeconomic effects of osteoporosis. A patient with osteoporosis has a 40% lifetime fracture risk, and most of them occur in the spine, hip or wrist (5). Osteoporotic fractures affect 8.9 million people around the world per year. It is expected that osteoporotic hip fractures among elderly men will increase by 310% and 240% in women by 2050 (6, 7). Osteoporosis and its complications have become a worldwide health crisis.

As a recently identified regulated cell death (RCD), ferroptosis differs from apoptosis (8), necrosis (9), autophagy (10), pyroptosis (11), etc. The main characteristics of ferroptosis are iron-dependent lipid peroxidation and reactive oxygen species (ROS) accumulation (12, 13). It involves multiple signaling pathways and their regulators (14) and has been extensively studied in the treatment of cancers, such as renal carcinoma and leukemia (15, 16) and hepatocellular carcinoma (17). Ferroptosis has been observed to be associated with various noncancer diseases, such as neurological diseases (18, 19), viral infection, ischemia and reperfusion injury, and atherosclerosis (20). Interestingly, a number of studies have suggested that ferroptosis may be a promising research direction in the prevention and treatment of osteoporosis (21–23).

Bone mesenchymal stromal cells (BMSCs) are pluripotent mesenchymal cells that can differentiate into different lineages, such as osteoblasts and adipocytes (24). BMSCs play an important role in maintaining bone homeostasis. As a result, osteoporosis is always accompanied by the low osteogenic potential of circulating BMSCs (25), the two form a vicious cycle, exacerbating the progression of osteoporosis. The delicate balance between osteogenic and lipogenic differentiation of BMSCs will be disrupted during the onset of osteoporosis (26). Under certain conditions, ferroptosis can also occur during the process of directional differentiation of BMSCs (27). At present, some researchers have also studied the mechanisms related to ferroptosis and several signaling pathways had been confirmed to play a role in the process of ferroptosis related osteoporosis (28). It is the existing basic research that provides inspiration and guidance for this study. Despite this, the specific mechanisms and related signaling pathways of ferroptosis remain mysterious in osteoporosis.

Bioinformatics analysis of ferroptosis genes in primary osteoporosis has not been reported to date. Thus, we identified DEGs in BMSCs of primary osteoporosis and healthy patients from the GEO databases with the help of bioinformatics analysis. By intersecting these DEGs with the ferroptosis dataset, ferroptosis DEGs (Ferr-DEGs) were obtained. After that, protein–protein interactions (PPI) were used to predict potential interactive networks. Then, the key Ferr-DEGs we obtained were further verified by an experimental OVX model. We have discovered the potential functional role of ferroptosis in BMSCs for the first time and have laid a possible foundation for understanding the pathological mechanism of primary osteoporosis.

## Materials and methods

### Microarray data collection

Information on primary osteoporosis patients was downloaded from the Gene Expression Omnibus (GEO; http://www.ncbi.nlm.nih.gov/geo), a public database that collects gene sequencing results (29). We downloaded the raw data of GSE35958 uploaded by Benisch from GEO (30). Five samples of primary osteoporosis (GSM878104, GSM878105, GSM878106, GSM878107, GSM878108; op group) and four controls (GSM878100, GSM878101, GSM878102, GSM878103; ctrl group) in GSE35958 were collected. A detailed table of patients’ parameters can be seen in **Supplementary Table S1**.

### Datasets analysis

Bioconductor was used to download the annotated R 4.0.3 package, and R was used to convert the microarray probes to symbols. When multiple probes corresponded to one gene ID, the average value was taken for analysis. Principal component analysis (PCA) was used to determine the significant difference dimensions with a P value < 0.05 (31). Relative log expression (RLE) was used for quality control (32). We used Limma to identify the DEGs. P-Value <0.05 and |log2-fold change (FC)| >1 were considered statistically significant (33). In addition, the Ferroptosis Database (FerrDb; http://www.zhounan.org/ferrdb) provided us with a dataset containing 259 genes (34). Then, we intersected it with the DEGs of GSE35958 to obtain ferroptosis DEGs (Ferr-DEGs).

### Functional enrichment analysis and visualization

Metascape (http://metascape.org) was used to determine gene functions (35). Biological process (BP), cellular component (CC) and molecular function (MF) are the three major components of gene ontology (GO), which are used to describe gene functions and interactions (36).. The Kyoto Encyclopedia of Genes and Genomes (KEGG) Pathway Database is an extensive database for mapping pathway annotation results (37, 38). GO and KEGG analyses were performed to explore the functional roles of the Ferr-DEGs *via* Metascape, and Cytoscape (V3.7.2) was used for visualization (39, 40). We then chose molecular complex detection (MCODE) to identify the interconnected central genes. Different colors are used to represent each MCODE network and the close interactions between molecules. During the interaction enrichment analysis, the minimum and maximum sizes of the selection network were 3 and 500, respectively.

### Protein–protein interaction network analysis

Then, Ferr-DEGs were uploaded to STRING (https://string-db.org/) to predict and construct PPI networks with a confidence level of >0.4 (41, 42). The node degree of proteins analyzed by STRING was used for ranking. Cytoscape was used to visualize the network. Nodes represent genes, and edges represent connections between genes. Genes were ranked by size according to the betweenness centrality (BC) score analyzed by the CytoNCA plugin. The cytoHubba plugin was used to rank genes by depth of the color corresponding to the weighted score based upon maximal clique centrality (MCC) algorithms (43, 44).

### Identification of key Ferr-DEGs

Protein and gene expression profiles at the tissue level (high, medium, low, NA) were obtained from the Human Protein Atlas database (HPA, https://www.proteinatlas.org). After calculation, we regarded the top ranked genes of each algorithm as hub genes and then took the trend of tissue expression and intersected most of those algorithms as the 5 most likely key Ferr-DEGs.

### OVX model construction and cell culture

We selected twelve eight-week-old female C57BL/6 mice (provided by the Experimental Animal Center of Soochow University) for osteoporosis induction experiments  (45). Mice were randomly divided into two groups: the ovariectomy (OVX) and sham groups. Eight weeks after surgery, all the specimens of the left femurs were removed for microCT and histological experiments. The right femurs were removed for BMSCs collection. Blood was collected by eyeballs removing, and the serum was separated by centrifugation at 2,000 r/m for 20 min and frozen at -80°C for future analysis. All procedures and experiments were approved by the Animal Ethics Committee of the First Affiliated Hospital of Soochow University.

BMSCs were obtained from the right femurs according to the protocol (46). Cells were seeded in a 10-cm^2^ dish and incubated in a 37°C incubator with 5% CO_2_. BMSCs after three passages were used for subsequent experiments.

### Micro-CT analysis

Left femur samples were scanned with a SkyScan 1176 micro-CT (Aartselaar, Belgium) (n = 6/group) after fixation in 10% buffered formalin for 48 h. Three-dimensional (3D) histomorphometric images were constructed by using NRecon software (SkyScan micro-CT, Aartselaar, Belgium). Bone mineral density (BMD), bone volume (BV), trabecular number (Tb. N), bone volume per tissue volume (BV/TV) and trabecular separation (Tb.sp) were used to evaluate bone mass. The region of interest (ROI) began with 100 pieces below the femur growth plate, and 200 pieces (6 µm each) were read per sample.

### RT–PCR

Total cellular RNA was obtained from BMSCs using TRIzol reagent (Beyotime, China), reverse transcription and amplification were performed using qRT supermix and SYBR qPCR master mix (Vazyme, China) according to the protocol. RT–PCR was performed in a CFX96™ thermal cycler (Bio-Rad Laboratories, USA). GAPDH was used as an internal reference to calculate the relative mRNA expression level. The primers are listed below: forward 5′-3′: GGTTGTCTCCTGCGACTTCA, reverse 5′-3′: TGGTCCAGGGTTTCTTACTCC for *Gapdh*. Forward 5′-3′: CGCTGTGGCAGATTGTTATTAA, reverse 5′-3′: TTGATCTGAAGTCAGGAATCCC for *Sirt1*. Forward 5′-3′: ATGATGAAGTTCACTGTGGTGG, reverse 5′-3′: CTGATCGTTGGCTATGATCTCC for *Hspa5*. Forward 5′-3′: CTGATCCTCAACGAGCTAGTTC, reverse 5′-3′: GGTCTTTGCAGTACTTGTCATG, for *Mtor*. Forward 5′-3′: GAATGAAGTGCACCCTAACAAG, reverse 5′-3′: GAGGAATGGGTTCACAAATCAG for *Hif1a*. Forward 5′-3′: TAATAGCTTCACTCTGATCGGG, reverse 5′-3′: CAAACAGCGTTTGTAGTTCTGA for *Becn1*.

### Lipid peroxidation assay

**A** Lipid Peroxidation Malondialdehyde (MDA) Assay Kit (Beyotime, China) was used to measure lipid peroxidation levels. Serum can be used directly for MDA detection. Cells were homogenized on ice in cell lysis buffer (Beyotime, China). After homogenization followed by centrifugation at 12,000×g for 10 minutes, the supernatant was collected for MDA assays. A 100 μl sample was added to each vial containing 200 μl of MDA detection working solution. The samples were mixed and incubated at 100°C for 15 mins. The samples were cooled to room temperature in a water bath and centrifuged at 1000×g for 10 minutes at room temperature. Then, 200 μl supernatant from each sample was pipetted into a 96-well plate, and the absorbance was measured at 532 nm with a microplate reader. MDA content was determined according to the standard curve.

### Iron assay

Serum and cellular iron levels were measured by an iron Content Assay Kit (Solarbio, China). Serum can be used directly for iron assay. 100μl serum was mixed with 250 μl of working solution and incubated at 100°C for 5 mins. The samples were cooled to room temperature in a water bath, mixed with 62 μl chloroform, and centrifuged at 10000×g for 10 minutes at room temperature. Then, 210 μl supernatant from each sample was pipetted into a 96-well plate, and the absorbance was measured at 520 nm. BMSCs were homogenized on ice in cell lysis buffer (Beyotime, China). After homogenization followed by centrifugation at 12,000×g for 10 minutes, 20 μl supernatant was collected and mixed with 180 μl working buffer at 25°C for 10 min, and then the absorbance was measured at 510 nm. A standard curve was simultaneously generated according to the manufacturer’s instructions.

### Immunofluorescence staining

We used immunofluorescence (IF) staining to detect the expression of Ferr-DEGs between the two groups. Antigen repair of the sections was performed, and the sections were blocked with horse serum. Finally, the primary antibody was incubated at 4°C overnight, and fluorescent-labeled secondary antibodies (ab150079, abcam, UK) were incubated at room temperature for 1 hour. The localization and protein expression level of *Becn1* (A7353, ABclonal, China) were observed by IF staining under an AxioCam HRC microscope (Carl Zeiss, Germany).

### Statistical analysis

The quantitative data are presented as the mean ± SD. Student’s t test was used to compare the differences between two groups. Each assay condition was performed in triplicate for all quantitative assays. A P value <0.05 was defined as statistically significant.

## Results

### Data quality control and identification of Ferr-DEGs

**Figure 1:**
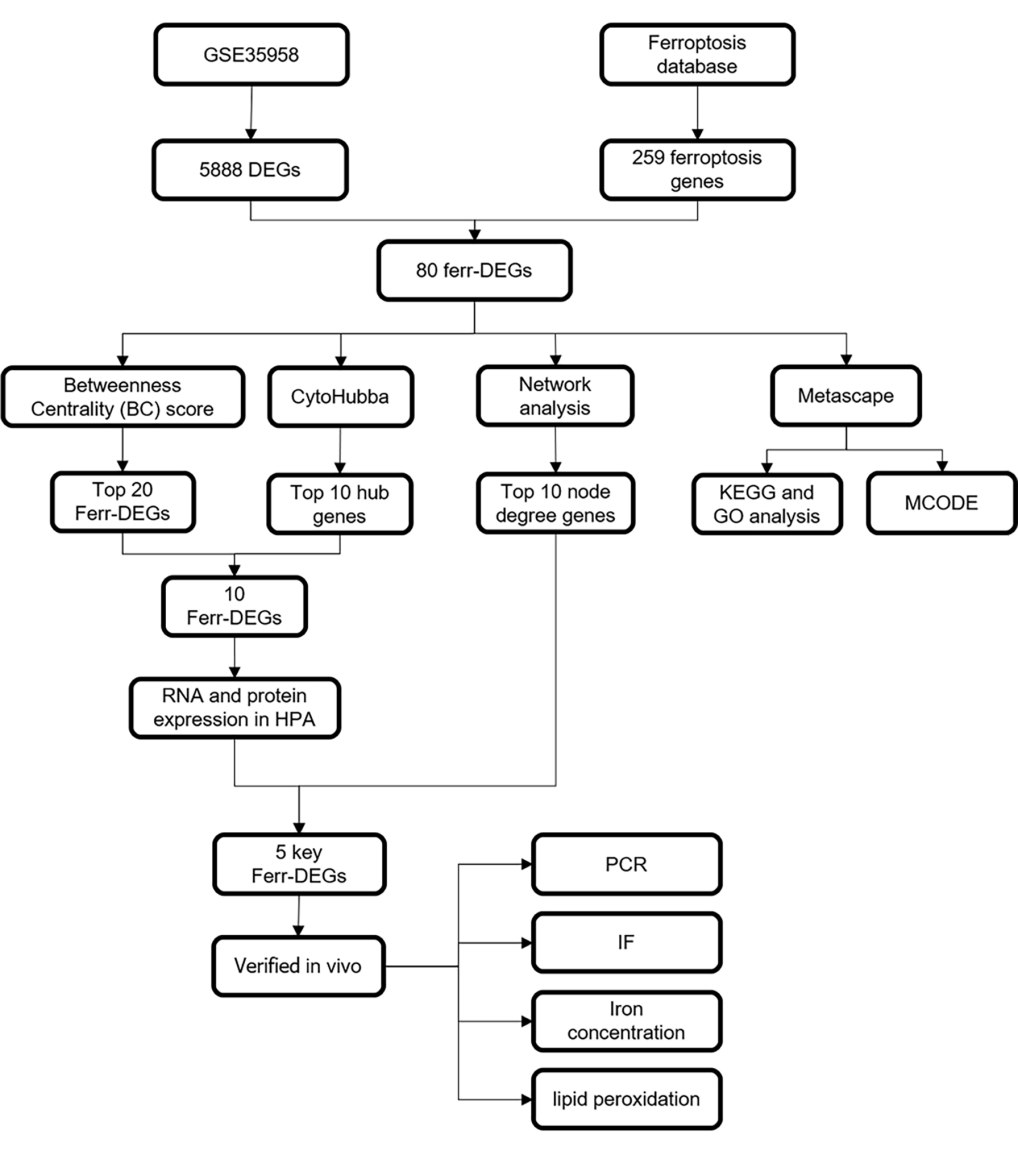
Schematic diagram showing the study design.

A schematic diagram of this study and the main findings is shown in **Figure 1**. Samples from GEO were divided into two groups [primary osteoporosis (OP) group versus control (Ctrl) group]. PCA and RLE analysis were performed to determine available dimensions and screen correlated samples according to quality control standards and the normalization of raw data. PCA demonstrated significant differences between the op group and the ctrl group (**Supplementary Figure S1A**). The RLE plots showed that the normalization was acceptable (**Supplementary Figure S1B**).

**Figure 2:**
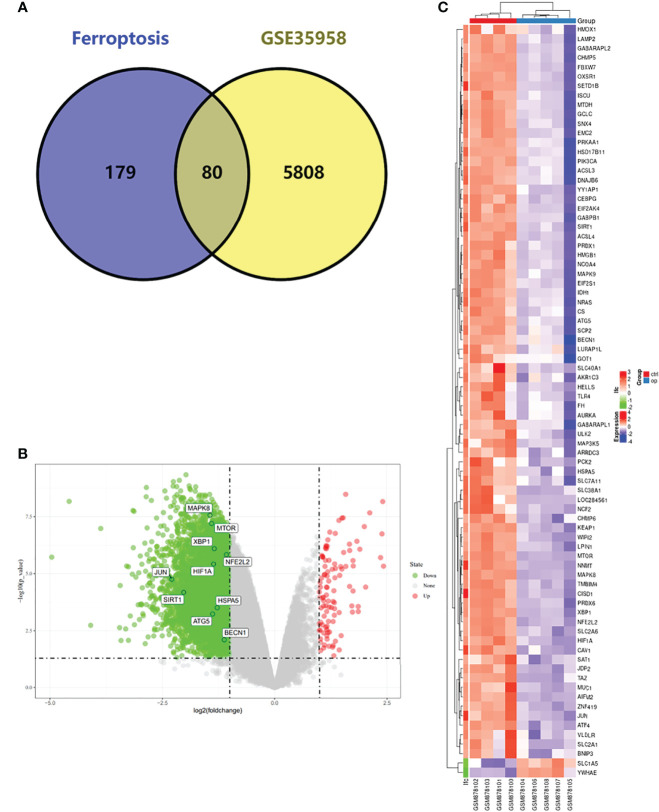
Detection of differentially expressed genes (DEGs) in the GSE35958 and Identification of shared DEGs with ferroptosis. **(A)** Venn diagram of DEGs in ferroptosis and GSE35958. **(B)** Volcano map of GSE35958, red represent up-regulated genes and green represent down-regulated genes, The top 10 genes identified by CytoHubba were marked in the diagram. **(C)** An expression heat map of the 80 Ferr-DEGs in GSE35958 dataset.

We first analyzed the DEGs of GSE35958 by using the “Limma” package and presented it in a volcano plot (**Figure 2B**) and heatmap (**Supplementary Figure S2**). Then, we obtained a gene set including 259 genes from FerrDb and intersected them with the DEGs of GSE35958 to identify Ferr-DEGs. Eighty Ferr-DEGs were found and are shown in a heatmap and Venn diagram (**Figures 2A, C**). The Ferr-DEGs were further divided into driver, suppressor and marker according to FerrDb (**Table 1**).

**Table 1:** Summary of the Ferr-DEGs.

Driver	Suppressor	Marker
*EMC2, PIK3CA, SCP2, ACSL4, TAZ, NRAS, SLC38A1, SLC1A5, GOT1, KEAP1, HMOX1, ATG5, NCOA4, IDH1, BECN1, GABARAPL2, SIRT1, DNAJB6, GABARAPL1, WIPI2, CS, SNX4, ULK2, SAT1, MAPK8, LPIN1, MAPK9, PRKAA1, HIF1A, HMGB1, TLR4, YY1AP1, MTDH, FBXW7*	*SLC7A11, AKR1C3, GCLC, NFE2L2, HMOX1, MUC1, SLC40A1, CISD1, HSPA5, ATF4, HELLS, MTOR, FH, ISCU, ACSL3, PRDX6, HIF1A, JUN, TMBIM4, AIFM2, LAMP2, CHMP5, CHMP6, CAV1*	*NCF2, BNIP3, OXSR1, SLC7A11, LOC284561, VLDLR, LURAP1L, XBP1, ZNF419, ARRDC3, JDP2, AURKA, CEBPG, EIF2S1, PRDX1, PCK2, GABPB1, HSD17B11, SETD1B, HMOX1, SLC40A1, ATF4, NFE2L2, MAP3K5, NNMT, SLC2A1, SLC2A6, IF2AK4, HMGB1, YWHAE*,

### Enrichment analysis of Ferr-DEGs

**Figure 3:**
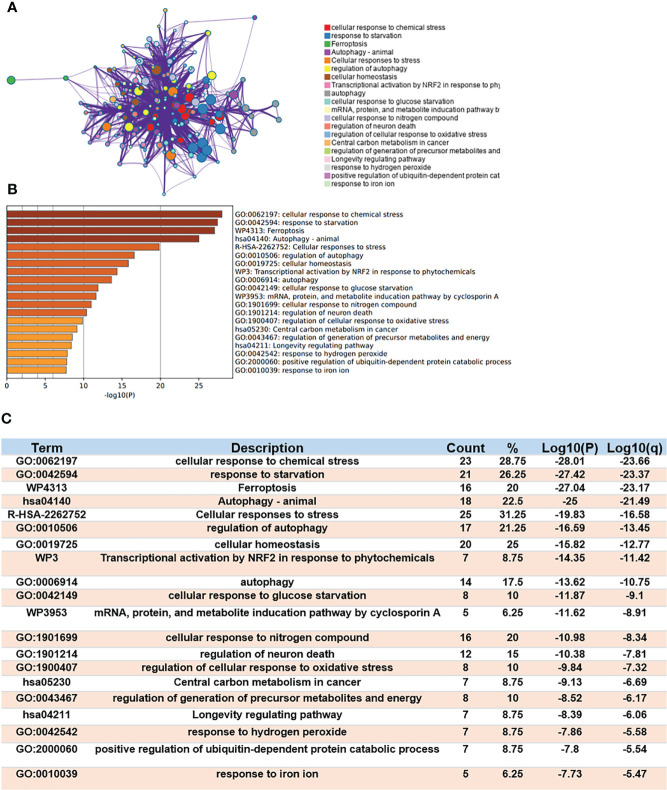
Functional enrichment analysis of DEGs. **(A)** Network of enriched terms. **(B)** A bar chart of top 20 biological pathways based on the P-value and the percentage of genes. **(C)** Details of top 20 biological pathways were listed in table.

Gene Ontology (GO) enrichment and Kyoto Encyclopedia of Genes and Genomes (KEGG) Pathway Database were used to analyze the biological classification of Ferr-DEGs by using Meyascape. The respective results of BP, CC, MF and KEGG are shown in detail (**Supplementary Figure S3**). The results showed that the significantly enriched genes were involved in the cellular response to stress, response to starvation, ferroptosis, autophagy, response to iron ion, etc. To further understand the relationships between different terms, we visualized it as a network plot (**Figures 3A, B**). Details of the top 20 terms are listed in the table (**Figure 3C**).

**Figure 4:**
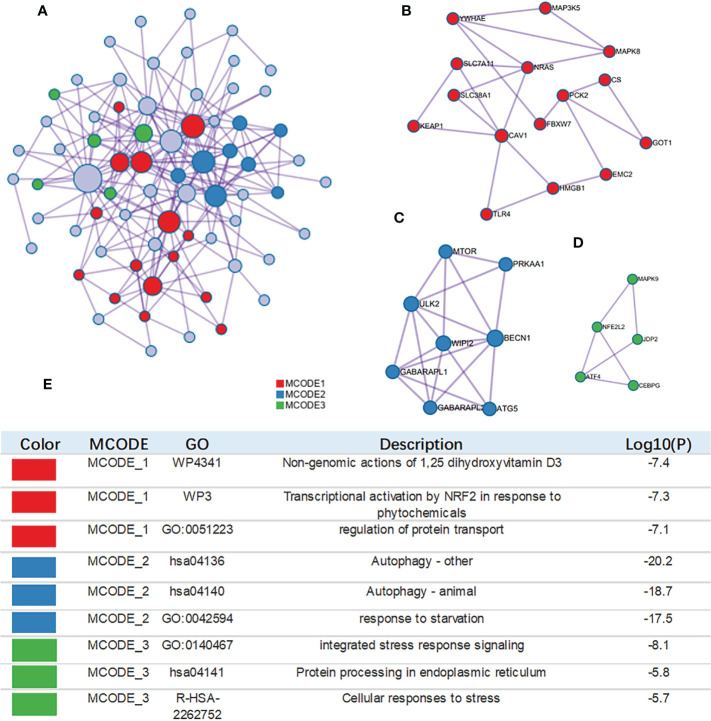
Construction of interactive network. **(A)** GO enrichment analysis was applied to each MCODE network. The same color nodes represent an interactive network and perform similar biological functions. **(B–D)** Three MCODE components were constructed with the screened hub genes. **(E)** Details of the 3 clusters were shown in table.

The MCODE algorithm was applied to explore those highly connected modules, and three of them were found (**Figures 4A–D**), The genes in Module 1 were associated with nongenomic actions of 1,25 dihydroxyvitamin D3, transcriptional activation by NFE2 like bZIP transcription factor 2(*NFE2L2*) in response to phytochemicals and regulation of protein transport. The genes in Module 2 were associated with autophagy and the response to starvation. The genes in Module 3 were related to protein processing and cellular responses to stress (**Figure 4E**).

### PPI network analysis and construction of Ferr-DEGs

**Figure 5:**
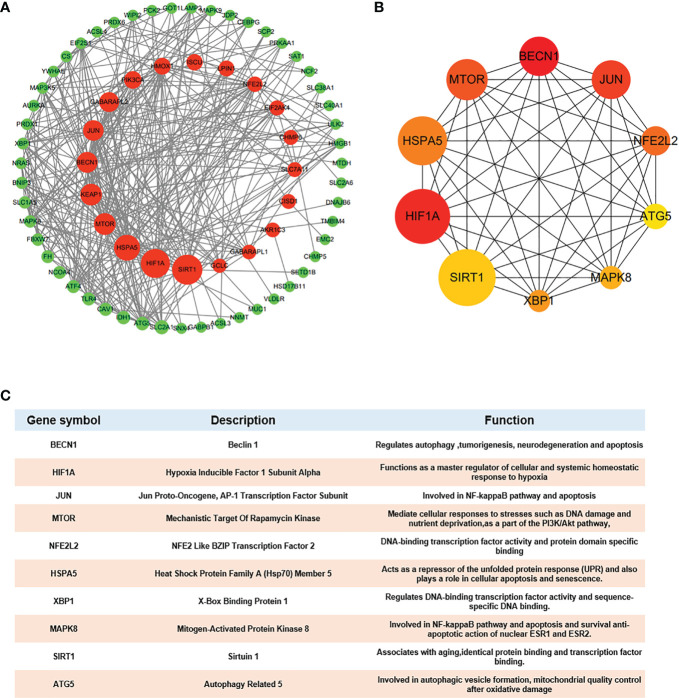
Hub gene identification **(A)** DEG PPI network constructed using Cytoscape, top 20 genes were shown inside the circle, genes were ranked by size according to Betweenness Centrality (BC) score. **(B)** Top 10 genes with the highest degree values were identified using CytoHubba and the depth of the color correspond to the weighted score. **(C)** The description and function of 10 hub genes were shown in table.

Because one of the 80 genes was not related to other molecules and did not form a molecular network, we obtained a PPI network containing 79 nodes and 348 edges. Node degree of proteins analyzed by STRING was used to rank, and the top 10 genes were mechanistic target of rapamycin kinase (*MTOR*), hypoxia inducible factor 1 subunit alpha (*HIF1A*), beclin 1 (*BECN1*), sirtuin 1(*SIRT1*), heat shock protein family A (Hsp70) member 5 (*HSPA5*), Jun proto-oncogene (*JUN*), *NFE2L2*, kelch like ECH associated protein 1(*KEAP1*), autophagy related 5 (*ATG5*) and heme oxygenase 1 (*HMOX1*) (details in **Supplementary Table S2**). Then, Cytoscape was used to visualize the network. The Ferr-DEG PPI network was constructed by CytoNCA. The top 20 genes are shown inside the circle, and the genes were ranked by size according to the betweenness centrality (BC) score (**Figure 5A**). Furthermore, we obtained the top 10 hub genes with the highest degree values by using CytoHubba (**Figure 5B**). Their descriptions and functions are shown in the table (**Figure 5C**).

### Selection and analysis of key Ferr-DEGs

**Figure 6:**
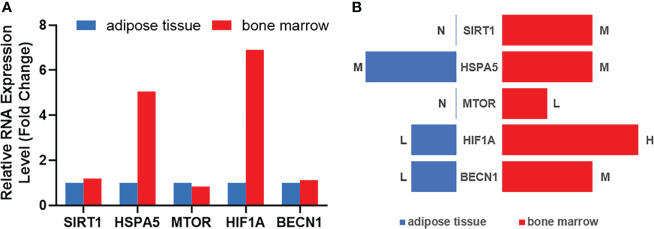
The expression analysis of hub genes in human tissues according to Human Protein Atlas data base. **(A)** Summary of RNA expression (fold change). **(B)** Summary of protein expression. H: high; M: medium; L: low; N: not detected.

We took the top gene in each algorithm as hub genes of different algorithms. After calculation, 10 Ferr-DEGs were obtained for further research. According to the literature, BMSCs tend to differentiate into adipogenic cells rather than osteogenic cells in osteoporosis patients. Therefore, protein and gene expression profiles at the tissue level (high, medium, low, NA) were obtained from HPA. We used the differences in their expression levels in adipose and bone marrow tissues as a preliminary reference for key Ferr-DEGs (**Supplementary Figure S4**). At the protein level, the expression of SIRT1, MAPK8*(Mitogen-activated protein kinase 8)*, NFE2L2, HIF1A and BECN1 in bone marrow was higher than that in adipose tissues. In contrast, the expression of ATG5 was higher in adipose tissues, and the expression level of HSPA5 was the same, while XBP1(X-box binding protein 1) and JUN were not detected in either tissue. At the gene level, the expression of *SIRT1*, *MAPK8*, *XBP1*, *HSPA5*, *HIF1A* and *BECN1* in bone marrow was higher than that in adipose tissues, and in contrast, the expression of *ATG5*, *NFE2L2*, *MTOR* and *JUN* was higher in adipose tissues. Finally, we considered the trends of protein level and gene expression in tissues and intersected most of those algorithms as the 5 most likely key Ferr-DEGs, including *SIRT1*, *HSPA5*, *MTOR*, *HIF1A* and *BECN1* (**Figures 6A, B**).

### Validation of the key Ferr-DEGs *in vivo*

**Figure 7:**
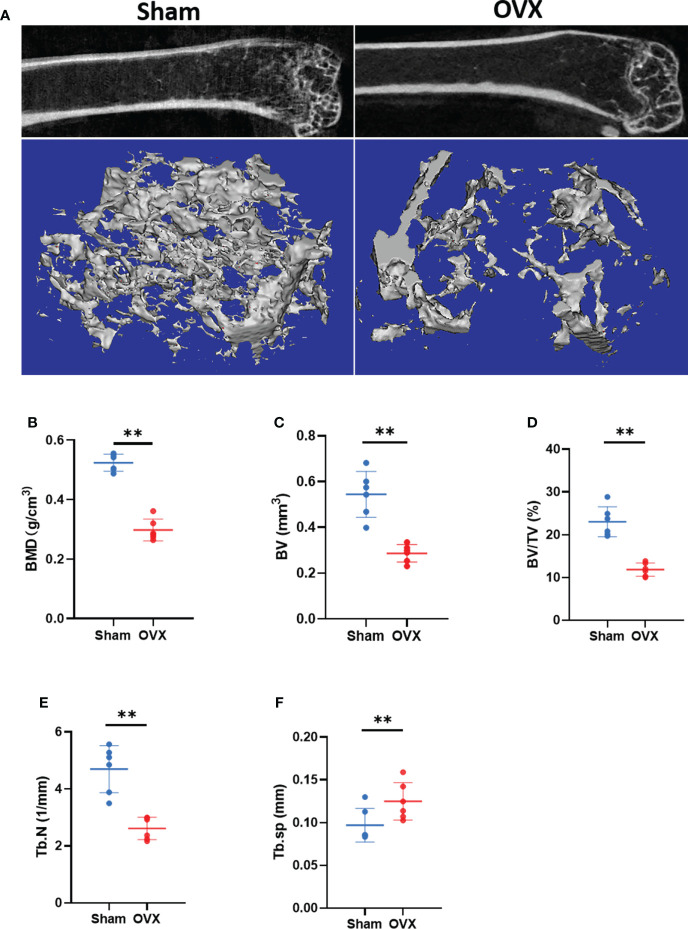
Micro CT results and analysis. **(A)** Representative 3D micro-CT reconstruction images of Sham and OVX group. **(B–F)** Quantitative analysis of bone parameters. (The data are expressed as the mean ± sd **P < 0.01).

**Figure 8:**
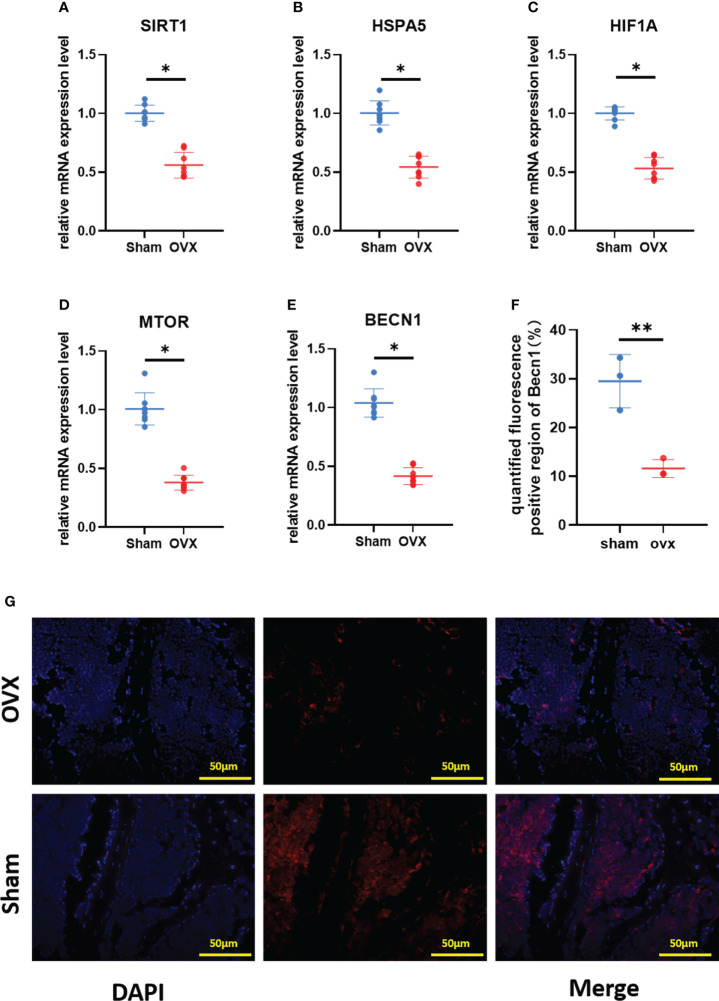
Validation of key Ferr-DEGs *in vivo*. **(A–E)** The mRNA expression level of Ferr-DEGs (*Sirt1*, *Hspa5*, *Mtor*, *Hif1a* and *Becn1*) in Sham and OVX group. **(F)** Quantitative analysis of fluorescence intensity. **(G)** Immunofluorescence staining of BECN1. (The data are expressed as the mean ± sd *P < 0.05, **P < 0.01).

To verify the key Ferr-DEGs *in vivo*, an OVX mouse model was used for validation. The OVX model was successfully established in C57BL/6 mice 8 weeks after surgery. The microCT results showed that the BMD, BV, BV/TV and Tb. N of the OVX group were decreased, while Tb.sp was increased (**Figure 7**). Meanwhile, the mRNAs of BMSCs isolated from the femurs were used to detect the expression of those 5 key Ferr-DEGs. The results showed that the relative mRNA expression levels of *Sirt1*, *Hspa5*, *Mtor*, *Hif1a* and *Becn1* in the OVX group were significantly lower than those in the sham group (**Figures 8A–E**). To further validate the expression of these five differentially expressed genes *in vivo*, we chose *Becn1* as the representative target, and IF staining was used for evaluation. The results showed that BECN1 was significantly more highly expressed in the sham group than in the OVX group (**Figures 8F, G**).

**Figure 9:**
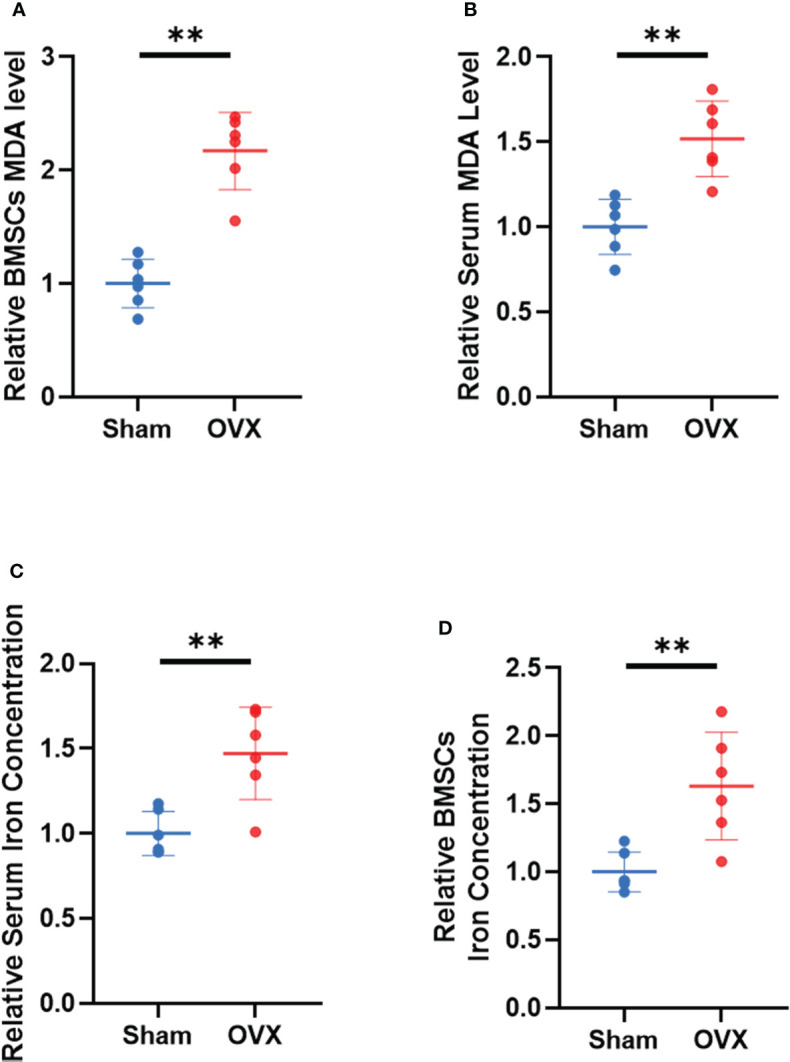
Validation of lipid peroxidation and iron level *in vivo*. **(A, B)** Relative expression of MDA in serum and BMSCs. **(C, D)** relative iron concentrations in serum and BMSCs. (The data are expressed as the mean ± sd **P < 0.01).

To further demonstrate the involvement of ferroptosis in the development of osteoporosis, lipid peroxidation levels and iron concentrations in serum and BMSCs from OVX mice were measured. The results showed that MDA levels and iron concentrations were significantly increased both in serum and BMSCs from OVX group (**Figures 9A–D**). Lipid peroxidation and iron overload are the characteristic manifestations of ferroptosis.

In summary, these *in vivo* results are consistent with the bioinformatics analysis, demonstrating that there is a high correlation between ferroptosis genes and the progression of osteoporosis in a mouse model. However, the exact mechanism needs to be further elucidated.

## Discussion

Different types of osteoporosis, such as diabetic osteoporosis, glucocorticoid-induced osteoporosis, and postmenopausal osteoporosis, were confirmed to be associated with ferroptosis (22, 47–50). However, the specific mechanisms and signaling pathways of ferroptosis involved in osteoporosis remain a mystery. We first intersected microarrays on primary osteoporosis patients in GEO with FerrDb, especially the detection and analysis of BMSCs, which has not been reported in previous studies. We aimed to identify the key genes of ferroptosis and explore its mechanisms in primary osteoporosis. Therefore, we compared the gene expression of primary osteoporosis patients with controls. To be more statistically persuasive, several bioinformatics algorithms (PCA, RLE, Limma, BC, MCC, etc.) were adopted to integrate the results. Additionally, PPI network construction, GO and KEGG were performed to explore the potential functional roles of the Ferr-DEGs. Finally, 80 Ferr-DEGs and 5 key Ferr-DEGs were calculated for further study.

As a newly discovered form of RCD, ferroptosis is accompanied by iron overload, lipid peroxidation and oxidative stress and is regulated by manifold genes. There appears to be a synergistic or antagonistic effect between ferroptosis and other RCDs because they have cross regulators in some pathways (51–53). Interestingly, according to our findings in this research, autophagy was regarded as the most crucial biological process after ferroptosis. Autophagy is an intracellular degradation system that maintains the stability of the intracellular environment (54, 55). Including three subtypes: microautophagy, macroautophagy and chaperone-mediated autophagy (CMA) (56–58). Several stress-related proteins were reported to be important regulators of autophagy-dependent ferroptosis in cancer therapy (59). New evidence suggests that abnormal levels of autophagy and mitochondrial autophagy disrupt the balance of bone metabolism. Osteoporotic BMSCs exhibit impaired osteogenic and increased adipogenic differentiation due to decreased autophagy (60, 61). Our results indicate that ferroptosis is involved in the process of osteoporosis and that autophagy is also involved. This conclusion was further supported by the MCODE results, which suggested that the Ferr-DEGs in different modules were connected with nongenomic actions of 1,25-dihydroxyvitamin D3, autophagy, and response to starvation and stress. We verified the potential connection between ferroptosis and autophagy in osteoporosis *via* various bioinformatics methods. The interaction between ferroptosis and autophagy deserves further study.

As a group of cells with the potential for multidirectional differentiation, BMSCs can differentiate into osteoblasts, chondrocytes and adipocytes. There is an opposite relationship between the differentiation of BMSCs into osteoblasts and adipocytes (62, 63). When osteoporosis occurs, BMSCs tend to differentiate into adipocytes rather than osteoblasts (64, 65). To test and verify our hypothesis, based on the HPA database, we compared the protein and gene expression levels of 10 hub genes between human bone marrow and adipose tissue. We considered the trends of protein and gene expression in tissues and intersected most of those algorithms as the 5 most likely key Ferr-DEGs, including *SIRT1*, *HSPA5*, *MTOR*, *HIF1A* and *BECN1*. Ultimately, these 5 key Ferr-DEGs were considered to be a preliminary reference for determining whether they are differentially expressed when osteoporosis occurs.

*SIRT1* is an important regulator of bone homeostasis. The results of HPA showed that the expression level of SIRT1 in adipose tissue is NA, which is not entirely consistent with what is known thus far as SIRT1 is a potential regulator in adipose tissue inflammation and metabolism. The conflicting results may be due to delays in updating the site. However, the relative expression levels of SIRT1 in adipose tissue and bone marrow tissue were consistent, which did not affect the conclusion of this study. *Sirt1* KO mice presented with osteoporosis characterized by decreased osteogenesis and increased adipogenesis in BMSCs (66). In contrast, BMSCs overexpressing *Sirt1* repressed the increased expression of superoxide dismutase 2 (*Sod2*) and forkhead box O3A (*Foxo3a*), which promoted the differentiation of BMSCs into osteoblasts and delayed senescence (67). A study in head and neck cancer cells showed that silencing *SIRT1* expression can inhibit epithelial-mesenchymal transition and decrease ferroptosis, while *SIRT1* agonists can promote ferroptosis (68). *SIRT1* was also reported to be associated with mitophagy (69). *HSPA5* is a chaperone protein mainly expressed in the Endoplasmic reticulum (ER) that maintains the stability of glutathione peroxidase 4 (*GPX4*) by forming the *HSPA5*-*GPX4* complex, thereby causing resistance to ferroptosis (70, 71). There seems to be no current research on the potential role of *HSPA5* in osteoporosis, which would be an interesting direction. *HIF1A* degradation, circadian rhythm and lipid peroxidation play a role in the regulation of ferroptosis, and *HIF1A* is a key factor (72). Feng et al. reported that ferroptosis might enhance diabetic nephropathy and damage renal tubules in diabetic mouse models (73). In orthopedic-related fields, it has been reported that *Hif1a*-dependent *Bcl2* interacting protein 3 (*Bnip3*) expression is increased and participates in hypoxia-induced autophagy activation, ultimately leading to osteoclastogenesis (74). The *Hif1a*-specific inhibitor 2ME2 can prevent osteoporosis in OVX mice, and the induction of ferroptosis by targeting *Hif1a* in osteoclasts may be a novel approach for the treatment of osteoporosis (50). Most studies on *BECN1* in ferroptosis are based on autophagy. *AMPK*-mediated *BECN1* phosphorylation can promote ferroptosis (75). The process of ferroptosis after erastin treatment was blocked by knockdown of *BECN1*, suggesting that ferroptosis may be a type of autophagy-dependent RCD (76). The osteogenic capability of an osteoblastic cell line was significantly reduced after knockdown of autophagy related 7 (*Atg7*) and *Becn1 (* 77). Our previous study also found that tet methylcytosine dioxygenase 2(*Tet2*) promoted bone loss in OVX mice. It positively regulates BECN1-dependent autophagy by inhibiting BCL2 expression and promoting osteoclast differentiation (78). However, whether *BECN1* is involved in ferroptosis during BMSCs differentiation is still unclear and will be further researched. Acts as a central regulator of the cellular response to growth stimulation, *MTOR* can inhibit autophagy-dependent ferroptosis. In cancer cells, interactions between *MTOR* and *GPX4* can regulate autophagy-dependent ferroptosis by inhibiting CMA (79), and *MTOR* inhibitors may promote *GPX4* degradation by activating the CMA pathway. *MTOR* also has a protective effect on ferroptosis in BMSCs, and autophagy mediated by the *MTOR* pathway can regulate the regeneration function of BMSCs, thereby influencing the occurrence and development of osteoporosis in postmenopausal women (61). Collectively, there are few studies about the specific functions and related mechanisms of these 5 key Ferr-DEGs in ferroptosis during osteoporosis progression. Our results provide a very meaningful direction for future research.

To further verify our conclusions, an osteoporotic model was successfully established in OVX mice. We found that the expression of *Sirt1*, *Hspa5*, *Mtor*, *Hif1a* and *Becn1* in BMSCs from the OVX group was lower than that in BMSCs from the control group, which is consistent with our bioinformatic analysis results. The results of immunofluorescence staining were also consistent with our expectations. Detection of lipid peroxidation and iron levels at serum and cellular levels also confirmed that ferroptosis may be involved in the development of primary osteoporosis. These outcomes suggest that ferroptosis of BMSCs may be involved in the pathological process of primary osteoporosis. Finally, we believe that our results will offer new insight into the role of ferroptosis in primary osteoporosis and identify the 5 key Ferr-DEGs as potential biomarkers for primary osteoporosis diagnosis and treatment.

## Conclusion

In summary, through a variety of bioinformatics methods, our research successfully identified 5 key Ferr-DEGs associated with primary osteoporosis and ferroptosis, namely, *SIRT1*, *HSPA5*, *MTOR*, *HIF1A* and *BECN1*. In addition, autophagy may also be involved in ferroptosis-related primary osteoporosis. In the future, we intend to collect enough bone tissues from patients with primary osteoporosis for further research. We aimed to clarify the regulatory roles of these genes to explore the differentiation mechanisms of BMSCs.

## Data availability statement

The datasets presented in this study can be found in online repositories. The names of the repository/repositories and accession number(s) can be found in the article/**Supplementary Material**.

## Ethics statement

The animal study was reviewed and approved by Animal Ethics Committee of the First Affiliated Hospital of Soochow University.

## Author contributions

YX and HZ: conception and design. HW and PZ: Acquisition and analysis of data. Investigation and software operation: QW. Validation: YX and YG. Writing and revising: YX and DG. Funding: DG and HY. All authors contributed to the article and approved the submitted version.

## Funding

This study was supported by grants from the National Natural Science Foundation of China (Nos. 82072425, 82072498, 81873991, 81873990, 81672238 and 81472077), the Young Medical Talents of Jiangsu Province (No. QNRC2016751), the Natural Science Foundation of Jiangsu Province (Nos. BK20180001 and BE2021650), and the Priority Academic Program Development of Jiangsu Higher Education Institutions (PAPD) and Special Project of Diagnosis and Treatment Technology for Key Clinical Diseases in Suzhou (LCZX202003).

## Conflict of interest

The authors declare that the research was conducted in the absence of any commercial or financial relationships that could be construed as a potential conflict of interest.

## Publisher’s note

All claims expressed in this article are solely those of the authors and do not necessarily represent those of their affiliated organizations, or those of the publisher, the editors and the reviewers. Any product that may be evaluated in this article, or claim that may be made by its manufacturer, is not guaranteed or endorsed by the publisher.

## Supplementary material

Supplementary Figure 1 Principal component analysis (PCA) and Relative log expression (RLE) plots of gene chips **(A)** PCA plot of GSE35958. Red and blue spots represent samples from op group and ctrl group respectively. **(B)** RLE plots of GSE35958.

Supplementary Figure 2 expression heat map of DEGs in GSE35958 dataset.

Supplementary Figure 3 GO and KEGG enrichment analysis. **(A–C)** A histogram of GO enrichment analysis of DEGs, included biological process, cellular component and molecular function. **(D)** Enrichment KEGG pathways.

Supplementary Figure 4 RNA and protein expression of top 10 hub genes in different human tissues. **(A)** Relative Rna expression level. **(B)** Summary of protein expression (H: high; M: medium; L: low; N: not detected)

## Abbreviations

BC, Betweenness Centrality; BP, Biological Process; BMSCs, Bone Mesenchymal Stromal Cells; BMD, Bone Mineral Density; BV, Bone Volume; BV/TV, Bone Volume Per Tissue Volume; CC, Cellular Component; CMA, Chaperone-Mediated Autophagy; DEGs, Differentially Expressed Genes; ER, Endoplasmic Reticulum; Ferr-DEGs, Ferroptosis DEGs; GEO, Gene Expression Omnibus; GO, Gene Ontology; HPA, Human Protein Atlas Database; IF, Immunofluorescence; KEGG, Kyoto Encyclopedia of Genes and Genomes; MCC, Maximal Clique Centrality; MCODE, Molecular Complex Detection; MF, Molecular Function; OVX, Ovariectomy; PCA, Principal Component Analysis; PPI, Protein–Protein Interactions; ROS, Reactive Oxygen Species; ROI, Region of Interest; RCD, Regulated Cell Death; RLE, Relative Log Expression; Tb. N, Trabecular Number; Tb.Sp, Trabecular Separation.
