# Supplementary figures and images for: Identification and validation of ferroptosis key genes in bone mesenchymal stromal cells of primary osteoporosis based on bioinformatics analysis
Source: Front Endocrinol (Lausanne). 2022 Aug 25;13:980867. doi: 10.3389/fendo.2022.980867 (PMC9452779; doi:10.3389/fendo.2022.980867)

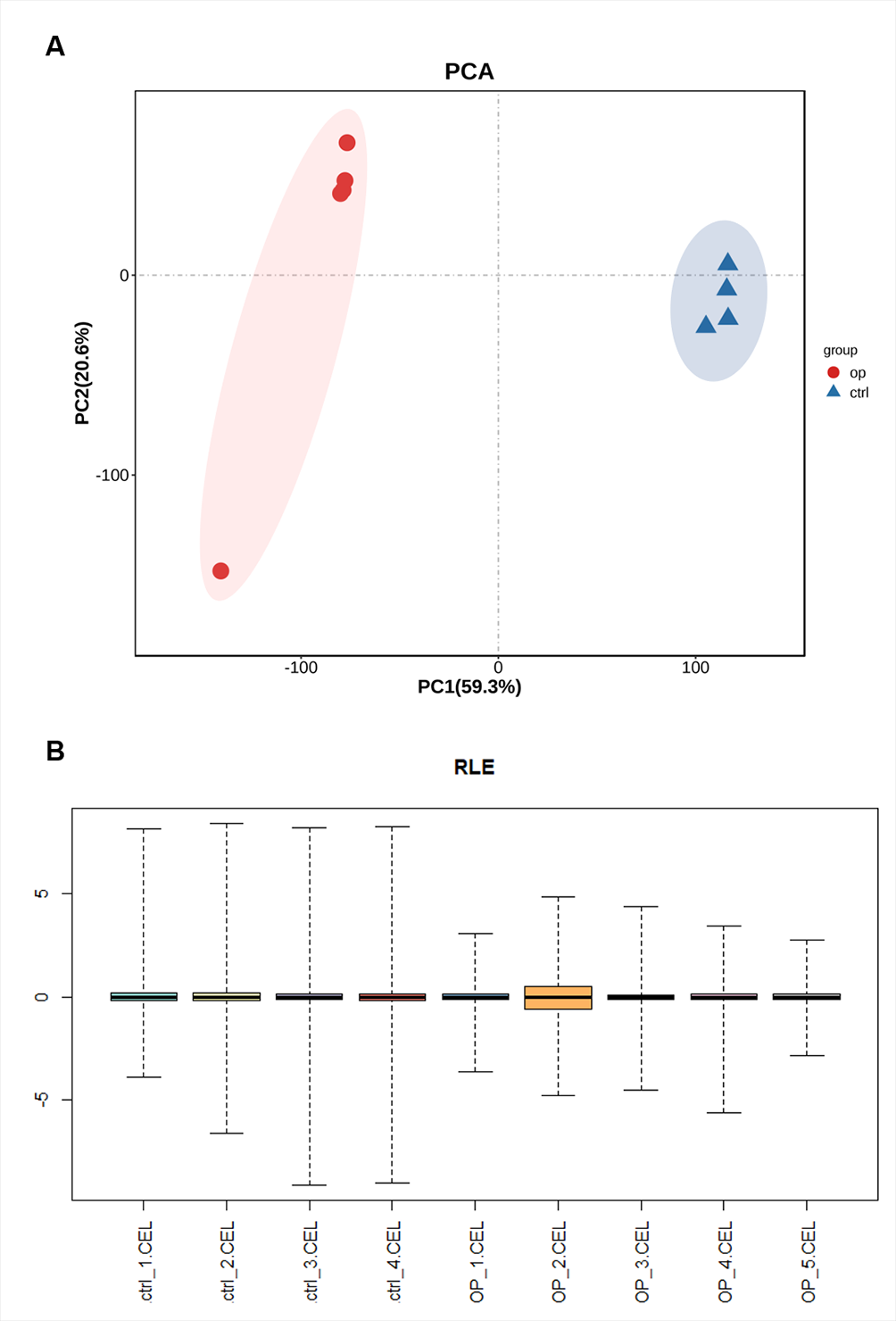

Supplement: Supplementary Figure 1 — Principal component analysis (PCA) and Relative log expression (RLE) plots of gene chips (A) PCA plot of GSE35958. Red and blue spots represent samples from op group and ctrl group respectively. (B) RLE plots of GSE35958. [file Image_1.tif]

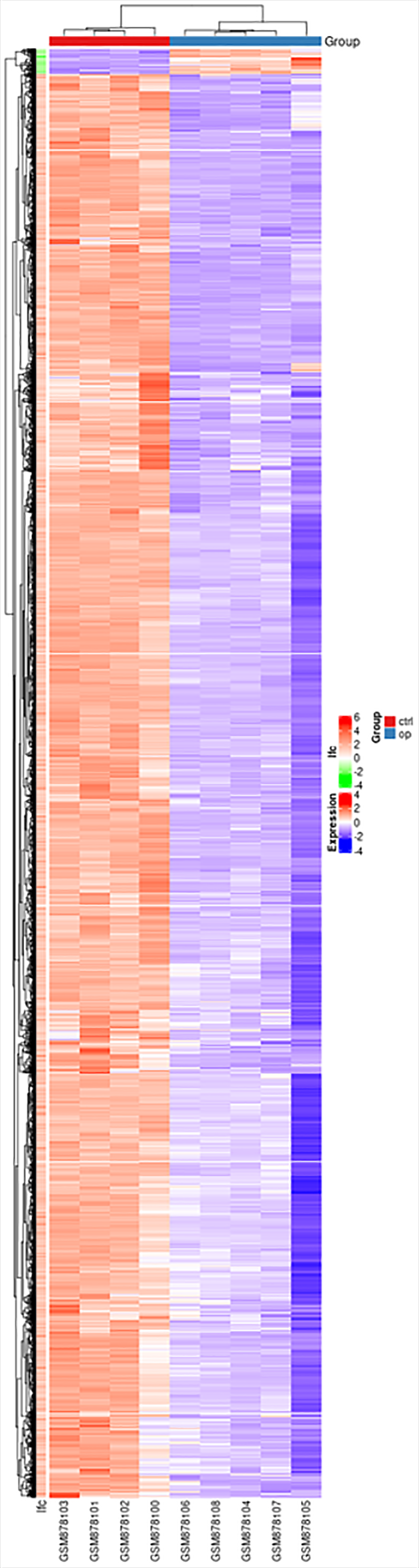

Supplement: Supplementary Figure 2 — expression heat map of DEGs in GSE35958 dataset. [file Image_2.tif]

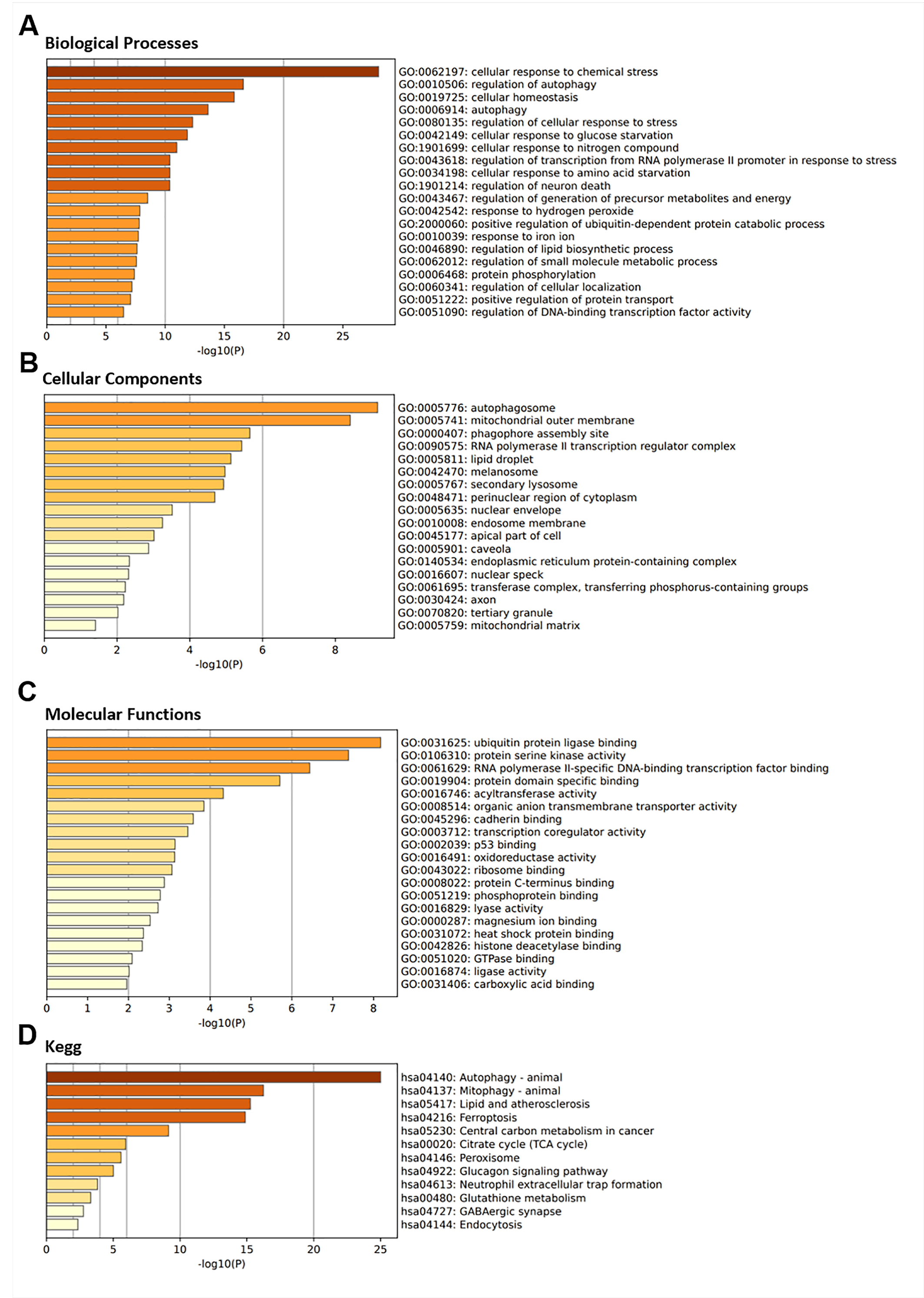

Supplement: Supplementary Figure 3 — GO and KEGG enrichment analysis. (A–C) A histogram of GO enrichment analysis of DEGs, included biological process, cellular component and molecular function. (D) Enrichment KEGG pathways. [file Image_3.tif]

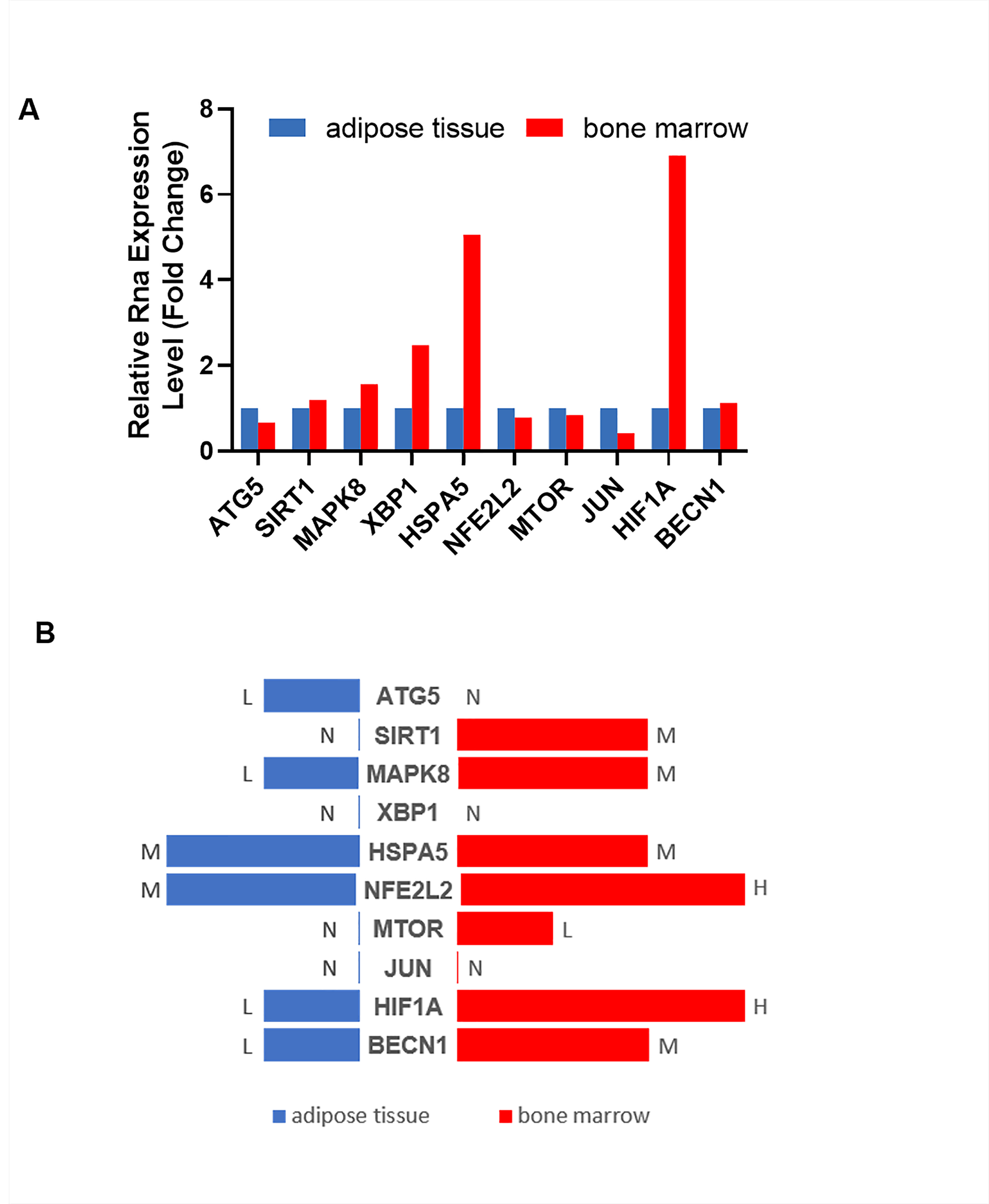

Supplement: Supplementary Figure 4 — RNA and protein expression of top 10 hub genes in different human tissues. (A) Relative Rna expression level. (B) Summary of protein expression (H: high; M: medium; L: low; N: not detected) [file Image_4.tif]
