# Supplementary material for: Identification and validation of ferroptosis key genes in bone mesenchymal stromal cells of primary osteoporosis based on bioinformatics analysis
Source: Front Endocrinol (Lausanne). 2022 Aug 25;13:980867. doi: 10.3389/fendo.2022.980867 (PMC9452779; doi:10.3389/fendo.2022.980867)
Supplement: Supplementary file 5 [file Table_1.docx]

Table s1 Summary of patients’ parameters

| Group | osteoporosis group | control group |
| --- | --- | --- |
| Donors | 5 | 4 |
| Average age (years) | 86.2±5.9 | 81.7±4.9 |
| gender | 5*female | 3*female+1*male |
